# Comparison of Official and Friendly Matches through Acceleration, Deceleration and Metabolic Power Measures: A Full-Season Study in Professional Soccer Players

**DOI:** 10.3390/ijerph18115980

**Published:** 2021-06-02

**Authors:** Hadi Nobari, Sara Mahmoudzadeh Khalili, Rafael Oliveira, Alfonso Castillo-Rodríguez, Jorge Pérez-Gómez, Luca Paolo Ardigò

**Affiliations:** 1 Department of Physical Education and Sports, University of Granada, 18010 Granada, Spain; acastillo@ugr.es; 2 HEME Research Group, Faculty of Sport Sciences, University of Extremadura, 10003 Cáceres, Spain; mahmoodzadesara@gmail.com (S.M.K.); jorgepg100@gmail.com (J.P.-G.); 3 Sports Scientist, Sepahan Football Club, Isfahan 81887-78473, Iran; 4 Department of Exercise Physiology, Faculty of Sport Sciences, University of Isfahan, Isfahan 81746-7344, Iran; 5 Department of Health and Sport Rehabilitation, Faculty of Sport Sciences and Health, Shahid Beheshti University, Tehran 198396-3113, Iran; 6 ESDRM-IPS-Sports Science School of Rio Maior—Polytechnic Institute of Santarém, 2040-413 Rio Maior, Portugal; rafaeloliveira@esdrm.ipsantarem.pt; 7 Research Centre in Sport Sciences, Health Sciences and Human Development, Quinta de Prados, Edifício Ciências de Desporto, 5001-801 Vila Real, Portugal; 8 Life Quality Research Centre, Complexo Andaluz, Apartado 279, 2001-904 Santarém, Portugal; 9 Department of Neurosciences, Biomedicine and Movement Sciences, School of Exercise and Sport Science, University of Verona, 37134 Verona, Italy

**Keywords:** acceleration, deceleration, friendly match, load monitoring, metabolic power, official match, performance

## Abstract

Soccer is a popular team sport and highly demanding activity that requires high effort and long-term training plans. The goals of this study were to compare the accelerations, decelerations and metabolic power between official and friendly full matches, between the first and second halves of the matches, and between both halves of official and friendly matches. Twelve professional soccer players (age, 28.6 ± 2.7 years; height, 182.1 ± 8.6 cm; body mass, 75.3 ± 8.2 kg; BMI, 22.6 ± 0.7 kg/m^2^) participated in this study. A total of 33 official and 10 friendly matches were analyzed from the Iranian Premier League. All matches were monitored using GPSPORTS systems Pty Ltd. The following variables were selected: total duration of the matches, metabolic power, accelerations Zone1 (<2 m·s^−2^) (AccZ1), accelerations Zone2 (2 to 4 m·s^−2^) (AccZ2), accelerations Zone3 (>4 m·s^−2^) (AccZ3), decelerations Zone1 (<−2 m·s^−2^) (DecZ1), decelerations Zone2 (−2 to −4 m·s^−2^) (DecZ2) and decelerations Zone3 (>−4 m·s^−2^) (DecZ3). The major finding was shown in metabolic power, where higher values occurred in friendly matches (*p* < 0.05 with small effect size). Furthermore, total duration, AccZ3, DecZ1, DecZ2, and DecZ3 were revealed to be higher in official matches, while AccZ1 and AccZ2 were higher in friendly matches. The second half of the official matches revealed higher values for total duration compared to friendly matches (*p* < 0.05, moderate effect size). In conclusion, this study observed higher values of metabolic power in friendly matches compared to official matches. AccZ3, DecZ1, DecZ2, and DecZ3 were higher in official matches, while AccZ1 and AccZ2 were higher in friendly matches.

## 1. Introduction

Soccer is a popular team sport and highly demanding activity that requires high effort and long-term training plans [1]. Therefore, high physical conditioning levels are needed to help soccer players to achieve specific training adaptations [2]. Moreover, to produce and maintain power during successive high-intensity efforts, soccer players need a high level of aerobic fitness [3]. It has been reported that during a match, elite soccer players cover about 9.1 km, where 1.3 km of the distance is performed at high-intensity running [4].

The main goal for friendly matches is to prepare the players/team for official matches; the results are thus not important, and friendly matches are not as competitive as the official matches. Therefore, during friendly matches, the psychological stress imposed on the players is less than during official matches. In friendly matches, the players cover about 10 km of distance [5]. Furthermore, the covered distance in the first half of the match is normally 5–10% greater than the distance covered in the second half [6,7]. In a competitive match, high-intensity acceleration and deceleration constitute a large part of the external biomechanical load [8]. Previous research used +3/−3 m per second squared (m·s^−2^) as a threshold of high/intense acceleration and deceleration, respectively [9,10], while recent studies [11,12] suggest that +2/−2 m·s^−2^ should be preferred to +3/−3 m·s^−2^ as a maximum threshold. The high-intensity decelerations are repeated 2.9 times more than high-intensity accelerations [13]. Professional players have the ability to maintain higher frequency and magnitude of acceleration or deceleration than novice players [14]. An investigation about the indicators of load-related injuries showed that the mechanical stress factors that appear in deceleration activities are important mediators of neuromuscular fatigue and tissue damage [15]. Likewise, it seems that acceleration demand is related to the players’ external load and should therefore be monitored continuously [16].

Acceleration (concentric muscular action) and deceleration (eccentric muscular action) are important factors in professional soccer. Acceleration is defined as the capacity to produce high speed in a short distance or time [17], while deceleration is the ability to reduce high velocity. There are three zones for both acceleration and deceleration: accelerations Zone1 (<2 m·s^−2^) (AccZ1); accelerations Zone2 (2 to 4 m·s^−2^) (AccZ2); accelerations Zone3 (>4 m·s^−2^) (AccZ3); decelerations Zone1 (<−2 m·s^−2^) (DecZ1); decelerations Zone2 (−2 to −4 m·s^−2^) (DecZ2); decelerations Zone3 (>−4 m·s^−2^) (DecZ3) [16,18]. During a match, accelerations contribute 7–10% and decelerations 5–7% of the players’ total load in all playing positions [19]. Evidently, within low to moderate intensity, more acceleration occurs than deceleration [19]. If the two halves of the match play are compared, the frequency of high- and very high-intensity accelerations and decelerations decreases from the first to the second half [8]. 

According to early measurements of metabolic demand, by assessing body temperature [20,21], it has been shown that the average metabolic load of a soccer player is 70% of the VO_2max_. Metabolic power has been presented as a tool to estimate the energy demands of movements with changeable speed, especially in team sports [16]. The concept of average metabolic power estimates the energy expenditure of acceleration and deceleration acquired by global positioning systems (GPS), and it seems that acceleration and deceleration are the main factors of energy cost [16]. The metabolic load is imposed on players during high-intensity levels of a match and also when acceleration is increased, even at a low speed. Metabolic power measures the energy expenditure, so it can be a good indicator of the average work intensity [16]. 

After FIFA allowed the use of electronic performance and tracking systems (EPTS) in competitive competitions in 2015, the use of GPS technology to track and control external loads in training and soccer matches has increased [22]. Measurement of acceleration, deceleration and metabolic power, derived from micro-technology devices like GPS, could be used in team sports to provide training load. It has been shown that GPS could be used within official competitive match play [23]. They are almost equal in size to a mobile phone, and can be carried by athletes during training sessions and matches [24]. It has been determined that GPS has the reliability and validity to monitor training [25]. Although it is stated that there are no changes between halves in the frequency of accelerations during elite matches [26], it is reported that acceleration and deceleration capacity are reduced in the second half [27]. Consequently, it is hypothesized that players might have different functions in friendly matches in comparison to official matches on acceleration, deceleration, and metabolic power averages. Moreover, these variables might have different values from the first to the second half. Given that monitoring the workload, including acceleration, deceleration, and metabolic power, can help to prevent training injuries, this information is very useful for coaches to design a suitable training program to achieve the goals of the team. Therefore, in this research, we used GPS to compare: firstly, accelerations, decelerations, and metabolic power between official and friendly matches; secondly, between the first and second halves of the games; and thirdly, the first and second halves between official and friendly matches, respectively, from the Iranian Premier League (IPL).

## 2. Materials and Methods

### 2.1. Experimental Approach to the Problem

This study included a professional soccer team that participated in the highest level of the IPL (Persian Gulf Premier League and knockout tournament in this country). In this league, each team is allowed to use GPS to record the physical fitness statistics of its players. We analyzed 33 official and 10 friendly matches.

During each match, players were monitored by a GPS (GPSPORTS systems Pty Ltd., Model: SPI High-Performance Unit (HPU); Fyshwick, Australian), and the study variables were collected daily during the full season (i.e., all training and matches). Details about how the GPS system was used are described in Section 2.3.

### 2.2. Participants

Twelve professional soccer players (age, 28.6 ± 2.7 years; height, 182.1 ± 8.6 cm; body mass, 75.3 ± 8.2 kg; BMI, 22.6 ± 0.7 kg/m^2^), who had at least eight years of experience training and competing in soccer league matches, participated in this study. The inclusion criterion required that players participate in at least three training sessions each week. Furthermore, the player had to participate in three consecutive full matches. The exclusion criteria included: (i) players with prolonged injury or a lack of participation in training for at least two consecutive weeks (two players were removed based on this criterion); (ii) goalkeepers were excluded from the study due to differences in training activities and workload in training and matches compared with field players. Table 1 presents the minutes played and running distances achieved by each player.

The experimental approach and study design were presented to the players after which written consent was obtained from all players. The study followed the ethical guidelines of the Helsinki Declaration for the study of humans, and was approved by the Ethics Committee of the University of Mohaghegh Ardabili (1395.10.20; decision date: 09.01.2017).

### 2.3. Monitoring External Workload

During the season, all workouts and match sessions were monitored using GPSPORTS systems Pty Ltd. These GPS-based tracking systems for professional athletes, model SPI HPU, include 15 Hz position GPS and data source body load (BL) using a triaxial accelerometer. According to a previous study, this device has high validity and reliability [18]. This unit was accurate for measuring high-sprinting velocities (coefficient of variation = 0.90%) [28]. There were no reported adverse weather conditions to affect data collection. 

Before starting the match, belts were placed on the players’ shoulders and chest based on previous studies [18,29,30,31]. After each cool-down session at the end of the training, the belts were collected from the players. All belts were checked by the team’s GPS manager and then entered into the dock system to download the information, which was then stored on the computer with the Team AMS software. The data from each session was automatically deleted from the belt memory after download. Before the next session, the belts were placed in an electric charging station. The SPI IQ Absolutes were adjusted for the GPS default zone throughout the season [32]. In addition, the personal characteristics (such as height and weight) of each player were entered into the software, and each player registered a belt with his name for use until the end of the season. The following variables were then selected: total duration of the matches, metabolic power, AccZ1 (<2 m·s^2^); AccZ2 (2 to 4 m·s^2^); AccZ3 (>4 m·s^2^); DecZ1 (<−2 m·s^2^); DecZ2 (−2 to −4 m·s^2^); DecZ3 (>−4 m·s^2^). According to the GPS manufacturer instructions, metabolic power calculation was based on previous research [16]. Moreover, metabolic power has a strong relationship with running distances [16] and for that reason, we added high-speed running distance and total sprint distance in Table 1. The comparison of these variables between official and friendly matches was done in the previous study [32].

### 2.4. Statistical Analysis

Data were analyzed using SPSS version 22.0 (SPSS Inc., Chicago, IL, USA) for Windows’ statistical software package. Initially, descriptive statistics were used to describe and characterize the sample. Shapiro−Wilk and Levene’s tests were used for the assumption of normality and homoscedasticity, respectively. *t*-tests with a 95% confidence interval (CI) were used to compare official vs. friendly matches and first vs. second halves, once variables obtained normal distribution (Shapiro−Wilk *p* > 0.05). The α level was set at *p* ≤ 0.05 for statistical significance. The effect-size statistic was calculated and expressed with a 95% CI to determine the magnitude of effects by standardizing the coefficients according to the appropriate between-subjects standard deviation, and it was assessed using the following criteria: <0.2 = trivial, 0.2 to 0.6 = small effect, >0.6 to 1.2 = moderate effect, >1.2 to 2.0 = large effect and >2.0 = very large [33]. 

In addition, *t*-test family sample power was calculated for a post-hoc compute achieve power (α level = 0.05 and *n* = 12) with the median effect size based on results by the G-Power [34]. There is an 85% (actual power) with the present analysis and sample. 

## 3. Results

Descriptive results and comparisons between official and friendly matches are presented in Table 2. Regarding full-match data, there was no difference between official and friendly matches for total duration of the matches. The major finding was found in metabolic power, where higher values occurred in friendly matches compared to official matches (*p* < 0.05 with small effect size). The other variables did not present significant differences. Furthermore, total duration, AccZ3, DecZ1, DecZ2, and DecZ3 were revealed to be higher in official matches, while AccZ1 and AccZ2 were higher in friendly matches. 

Regarding comparisons between the first half of official vs. friendly matches, there were no significant differences in all variables (all, *p* > 0.05). Regarding comparisons between the second half of official vs. friendly matches, there were higher values found for the duration (*p* < 0.05, moderate effect size), but the other variables did not present differences between official and friendly matches during the second half.

Comparisons between first and second halves for official and friendly matches are presented in Table 3. Regarding official matches, there were higher durations for AccZ1, AccZ2 AccZ3, DecZ1, DecZ2 in the first half of official matches (all, *p* < 0.05, moderate to large effect size). Regarding friendly matches, there were higher durations in AccZ1, AccZ3, DecZ1, DecZ2 during the first half of friendly matches (all, *p* < 0.05, moderate effect size).

## 4. Discussion 

The main goal of this study was to compare the acceleration, deceleration and metabolic power in official and friendly matches, and also to compare these variables between the two halves of the matches. 

Our hypotheses were that players might have different functions in friendly matches in comparison to official matches on acceleration, deceleration, and metabolic averages, and that these variables might have different values from the first to the second half. The study confirmed our hypotheses. The major finding was higher values of metabolic power in both halves of friendly matches (*p* < 0.05 with small effect size) compared to official matches. Metabolic power is an estimation tool for the energetic demands of variable-speed locomotion, which is very common in team sports. Osgnach et al. stated that metabolic power output at high intensity or sprinting is justly elevated. However, in low running speeds, whenever the acceleration is elevated, a similar power can also be achieved [12]. According to the data, AccZ1 and AccZ3 are higher in the first half, and AccZ2 is higher in the second half of the friendly match rather than the official match. Therefore, there might be a relationship between elevation of acceleration and increase in metabolic power.

Furthermore, AccZ3, DecZ1, DecZ2, and DecZ3 were revealed to be higher in the official match while AccZ1 and AccZ2 were higher in friendly matches. It has been stated that acceleration might be a sensitive measure for high-speed activities. Akenhead et al. reported that acceleration and deceleration capacity diminished during a soccer match [35]. The result of a study in Australian soccer showed a remarkable decrease in the number of maximal accelerations when the match progressed [36]. Freitas et al. stated that friendly matches do not require high-performance activity demands compared to official matches [37]. During official matches, due to the importance of the game, match intensity, competitive anxiety, and probably, higher level of athletes’ commitment, psychological pressure is applied to the players [37,38].

In this study, regarding comparisons between the second half of official vs. friendly matches, higher values were found for duration in official matches (*p* < 0.05, moderate effect size), but the other variables did not present differences between the second halves of official and friendly matches. Most of the external load variables showed higher values during the first few minutes of exercise [39,40]. Some studies revealed that during the second half, the distance run decreases [41,42]. Moreover, total distance and high-intensity running for players in a ‘high’ activity group were significantly lower in the second half [43]. Dalen et al. explained that acceleration in the second half was 14% less than the first half of the soccer match [44]. This decrement could result in a state of advanced fatigue in the last minutes of the second half [42]. However, Bradley et al. reported no changes between halves in the frequency of accelerations during elite matches when classified into two intensity thresholds (medium or high) [26]. In addition, Akenhead et al. showed that there is no change in the distances at high-intensity soccer matches, although other markers of acceleration and deceleration capacity illustrate small declines throughout the second half [35]. In this regard, Russell et al. expressed that although the number of accelerations and decelerations are reduced in the second half, the distance covered at high intensity remained steady between halves [27]. Unfortunately, the present study did not analyze that variable.

Despite the new findings of this study, there were some limitations. Soccer is a team sport that is dependent on different factors, and it is determined by the interaction of technical, tactical, physiological, and psychological components [45]. In this research, psychological factors were not considered, and it is recommended to investigate the effect of this factor in future studies. Another limitation of the present study was the lack of matching of tactical systems of teams and venue (i.e., home or away) of the competitions. In future studies, it is strongly recommended that researchers consider comparison between games based on tactical and venue matching. Moreover, match results were not considered for analysis which could influence the present findings, and for that reason, we suggest a further analysis for future studies. The sample size was twelve, which is a small size in comparison to similar articles [19,44]. It is suggested that to generalize the finding of this study, the number of participants should be increased. However, the strength of this study is the dedication to professional soccer players, and considering the two common types of soccer matches, official and friendly. The training and competition environment from this study also represents the real scenario during a full season. Furthermore, utilizing the data of GPS, an accurate and reliable microtechnology, is the other positive side of this research.

Considering the findings of this article, the use of GPS-based tracking systems, which include 15 Hz position GPS and data source BL using a triaxial accelerometer, might be useful to assess players’ total duration, acceleration, deceleration, and metabolic power in soccer match play. Additionally, considering that metabolic power is an estimation tool for the energetic demands of variable-speed locomotion and that it is elevated in both halves of friendly matches, it is better to be monitored. Therefore, coaches and practitioners can use the results of this research to modify training sessions and prepare players for different types of competition, according to the specific requirements of each match.

## 5. Conclusions

In conclusion, the major finding of this study was that higher values of metabolic power were found in friendly matches compared to official matches. Total duration was high in both halves of the official and friendly matches. Furthermore, AccZ3, DecZ1, DecZ2, and DecZ3 were higher in official matches while AccZ1 and AccZ2 were higher in friendly matches. 

## Figures and Tables

**Table 1:** Average and standard deviation of match duration and running distances by each player during official and friendly matches.

Players	Official Matches Duration (min)	Friendly Matches Duration (min)	HSRD (m) of Official Matches	HSRD (m) of Friendly Matches	Total Sprint Distance (m) of Official Matches	Total Sprint Distance (m) of Friendly Matches
1	97.2 ± 5.5	88.3 ± 23.7	113.5 ± 73.7	109.95 ± 89.3	21.4 ± 14.8	11.47 ± 6.1
2	65.6 ± 8.9	84.8 ± 27.4	280.5 ± 77.9	337.32 ± 135.4	25.1 ± 17.7	20.37 ± 14.9
3	96.2 ± 2.9	92.0 ± 1.9	192.1 ± 12.5	222.94 ± 71.0	40.9 ± 17.9	4.79 ± 28.8
4	92.0 ± 20.5	88.2 ± 16.3	264.4 ± 62.2	205.81 ± 173.3	17.4 ± 3.7	36.48 ± 15.1
5	97.5 ± 3.5	90.0 ± 28.6	121.8 ± 82.7	100.34 ± 100.6	10.6 ± 0	11.02 ± 0
6	86.5 ± 17.3	83.8 ± 25.5	333.6 ± 131.7	213.43 ± 143.4	49.4 ± 29.8	23.11 ± 25.6
7	89.5 ± 14.5	84.9 ± 13.8	285.5 ± 120.3	279.18 ± 174.5	16.4 ± 13.9	42.17 ± 13.2
8	74.7 ± 29.4	79.1 ± 13.5	211.6 ± 61.3	234.71 ± 120.5	25.0 ± 0	16.48 ± 5.6
9	94.5 ± 9.5	79.6 ± 11.6	382.0 ± 110.8	248.49 ± 181.2	47.6 ± 31.7	10.38 ± 19.4
10	97.4 ± 3.4	83.1 ± 24.3	304.8 ± 107.1	262.90 ± 102.7	37.5 ± 36.6	33.37 ± 23.95
11	69.1 ± 37.4	85.9 ± 9.9	231.4 ± 86.2	301.03 ± 148.4	24.6 ± 17.8	33.42 ± 21.0
12	94.8 ± 1.9	90.1 ± 13.1	178.7 ± 88.0	130.38 ± 76.5	39.0 ± 27.6	26.52 ± 12.1

HSRD, high-speed running distance (18–23 km·h^−1^); total sprint distance (>23 km·h^−1^); min, minutes; m, meters.

**Table 2:** Comparison of full match day, first half, and second half data between official and friendly matches per squad average, mean ± standard deviation.

Full match	Official Matches (CI, 95%)	Friendly Matches (CI, 95%)	*p*	CI (95%)	Effect Size
Duration (min)	87.9 ± 11.6 (80.5–95.3)	85.8 ± 4.1 (83.2–88.4)	0.514	−4.7, 8.9	0.24 (−0.57, 1.04)
Metabolic power (W·kg^−1^),	18.4 ± 2.0 (17.2–19.7)	19.5 ± 1.7 (18.4–20.5)	0.029 *	−2.0, −0.1	−0.59 (−1.39, 0.24)
AccZ1 (m·s^−2^)	126.1 ± 19.6 (113.6–138.5)	129.5 ± 22.4 (115.3–143.8)	0.616	−18.4, 11.4	−0.16 (−0.96, 0.65)
AccZ2 (m·s^−2^)	35.2 ± 6.4 (35.1–39.3)	36.7 ± 7.2 (32.1–41.3)	0.510	−6.1, 3.2	−0.41 (−1.20, 0.41)
AccZ3 (m·s^−2^)	4.4 ± 1.2 (3.6–5.2)	4.3 ± 1.2 (3.5–5.1)	0.849	−0.8, 1.0	−0.67 (−1.46, 0.18)
DecZ1 (m·s^−2^)	62.4 ± 10.5 (55.7–69.1)	62.2 ± 11.0 (55.2–69.2)	0.950	−6.0, 6.4	<0.001 (−0.80, 0.80)
DecZ2 (m·s^−2^)	23.2 ± 3.7 (20.8–25.5)	21.9 ± 6.3 (17.9–26.0)	0.490	−2.6, 5.0	−0.10 (0.89, 0.71)
DecZ3 (m·s^−2^)	8.4 ± 1.6 (7.5–9.5)	8.7 ± 2.2 (7.3–10.1)	0.753	−1.5, 1.1	−1.35 (−2.19, −0.42)
**1st half**	**Official Matches (CI, 95%)**	**Friendly Matches (CI, 95%)**	***p***	**CI (95%)**	**Effect Size**
Duration (min)	47.1 ± 1.8 (45.9–48.2)	47.2 ± 2.7 (45.5–48.9)	0.872	−1.9, 1.6	<0.001 (−0.80, 0.80)
Metabolic power (W·kg^−1^),	9.7 ± 0.8 (9.2–10.2)	9.8 ± 0.9 (9.3–10.4)	0.585	−0.5, 0.3	−1.06 (−1.87, −0.17)
AccZ1 (m·s^−2^)	71.1 ± 11.6 (63.7–78.4)	71.8 ± 12.8 (63.7–79.9)	0.837	−8.0, 6.6	−0.01 (−0.81, 0.79)
AccZ2 (m·s^−2^)	19.8 ± 4.4 (17.1–22.6)	19.4 ± 4.7 (16.4–22.5)	0.750	−2.3, 3.0	−0.26 (−1.06, 0.55)
AccZ3 (m·s^−2^)	2.5 ± 0.8 (1.9–3.0)	2.5 ± 0.9 (2.0–3.0)	0.847	−0.5, 0.5	−0.35 (−1.15, 0.47)
DecZ1 (m·s^−2^)	34.4 ± 6.8 (30.1–38.8)	34.3 ± 6.6 (30.1–38.4)	0.896	−2.7, 3.0	−0.01 (−0.81, 0.79)
DecZ2 (m·s^−2^)	13.0 ± 2.6 (11.3–14.6)	12.4 ± 4.7 (9.4–15.4)	0.604	−1.7, 2.9	0.05 (−0.75, 0.85)
DecZ3 (m·s^−2^)	4.7 ± 1.5 (3.7–5.7)	4.5 ± 1.6 (3.5–5.6)	0.769	−1.1, 1.4	−1.35 (−2.19, −0.43)
**2nd half**	**Official Matches (CI, 95%)**	**Friendly Matches (CI, 95%)**	***p***	**CI (95%)**	**Effect Size**
Duration (min),	43.4 ± 6.2 (39.4–47.3)	38.6 ± 4.2 (35.9–41.3)	0.016 *	1.1, 8.4	0.91 (0.04, 1.71)
Metabolic power (W·kg^−1^),	9.3 ± 1.1 (8.5–10.0)	9.6 ± 1.0 (9.0–10.3)	0.136	−0.9, 0.1	<0.001 (−0.80, 0.80)
AccZ1 (m·s^−2^)	58.9 ± 7.6 (54.1–63.7)	57.8 ± 12.0 (50.1–53.0)	0.771	−7.4, 9.7	0.01 (−0.79, 0.81)
AccZ2 (m·s^−2^)	16.6 ± 3.1 (14.7–18.6)	17.2 ± 3.8 (14.8–19.6)	0.625	−3.2, 2.0	−1.27 (−2.10, −0.35)
AccZ3 (m·s^−2^)	2.0 ± 0.5 (1.7–2.3)	1.8 ± 0.6 (1.4–2.2)	0.272	−0.2, 0.6	−1.27 (−2.10, −0.35)
DecZ1 (m·s^−2^)	30.0 ± 4.2 (27.4–32.7)	27.9 ± 5.2 (24.6–31.3)	0.168	−1.0, 5.2	−0.02 (−0.82, 0.78)
DecZ2 (m·s^−2^)	11.0 ± 1.5 (10.0–11.9)	9.5 ± 2.3 (8.0–11.0)	0.070	−0.1, 3.1	−0.52 (−1.31, 0.32)
DecZ3 (m·s^−2^)	4.1 ± 0.8 (3.6–4.7)	4.1 ± 1.1 (3.4–4.9)	0.982	−0.6, 0.6	−0.83 (−1.63, 0.03)

CI, confidence interval; AccZ1, accelerations in zone 1 (<2 m·s^−2^); AccZ2, accelerations in zone 2 (2 to 4 m·s^−2^); AccZ3, accelerations in zone 3 (>4 m·s^−2^); DecZ1, decelerations in zone 1 (>−2 m·s^−2^); DecZ2, decelerations in zone 2 (−2 to −4 m·s^−2^); DecZ3, decelerations in zone 3 (<−4 m·s^−2^); * significant differences between official match vs. friendly match, *p* < 0.05.

**Table 3:** Comparison of first and second halves during official and friendly matches.

Official Matches	*p* (1st half vs. 2nd half)	Confidence Interval (95%)	Effect Size
Duration (min), *n* = 10	0.034 *	0.3, 7.1	−1.38 (−2.22, −0.45)
Metabolic power (W·kg^−1^),	0.027 *	0.1, 0.8	0.42 (−0.41, 1.21)
AccZ1 (m·s^−2^)	0.002 *	5.4, 19.0	1.24 (0.33, 2.07)
AccZ2 (m·s^−2^)	0.016 *	0.7, 5.7	0.84 (−0.02, 1.64)
AccZ3 (m·s^−2^)	0.021 *	0.1, 0.8	0.75 (−0.10, 1.55)
DecZ1 (m·s^−2^)	0.022 *	0.8, 8.1	0.78 (−0.08, 1.58)
DecZ2 (m·s^−2^)	0.015 *	0.5, 3.5	0.94 (0.07, 1.75)
DecZ3 (m·s^−2^)	0.215	−0.4, 1.5	0.50 (−0.33, 1.29)
**Friendly Matches**	***p*** **(1st half vs. 2nd half)**	**Confidence Interval (95%)**	**Effect Size**
Duration (min), *n* = 13	<0.001 *	4.9, 12.3	2.44 (1.31, 3.39)
Metabolic power (W·kg^−1^),	0.463	−0.3, 0.7	<0.001 (−0.80, 0.80)
AccZ1 (m·s^−2^)	0.001 *	7.3, 20.7	1.13 (0.23, 1.95)
AccZ2 (m·s^−2^)	0.127	−0.7, 5.2	<0.001 (−0.80, 0.80)
AccZ3 (m·s^−2^)	0.023 *	0.1, 1.3	0.92 (0.05, 1.72)
DecZ1 (m·s^−2^)	0.001 *	3.5, 9.2	<0.001 (−0.80, 0.80)
DecZ2 (m·s^−2^)	0.023 *	0.5, 5.3	0.78 (−0.07, 1.59)
DecZ3 (m·s^−2^)	0.424	−0.6, 1.4	<0.001 (−0.80, 0.80)

AccZ1, accelerations in zone 1 (<2 m·s^−2^); AccZ2, accelerations in zone 2 (2 to 4 m·s^−2^); AccZ3, accelerations in zone 3 (>4 m·s^−2^); DecZ1, decelerations in zone 1 (>−2 m·s^−2^); DecZ2, decelerations in zone 2 (−2 to −4 m·s^−2^); DecZ3, decelerations in zone 3 (<−4 m·s^−2^); * denotes difference from 2nd half. All *p* < 0.05.

## Data Availability

The datasets used and/or analyzed during the current study are available from the corresponding author on reasonable request.
